# Bouncing Back: Plant-Associated Soil Microbes Respond Rapidly to Prairie Establishment

**DOI:** 10.1371/journal.pone.0115775

**Published:** 2014-12-31

**Authors:** Anna J. Herzberger, David S. Duncan, Randall D. Jackson

**Affiliations:** 1 Department of Fisheries and Wildlife, Michigan State University, East Lansing, United States of America; 2 Department of Agronomy, University of Wisconsin, Madison, United States of America; 3 DOE-Great Lakes Bioenergy Research Center, University of Wisconsin, Madison, United States of America; North Carolina State University, United States of America

## Abstract

It is well established that soil microbial communities change in response to altered land use and land cover, but less is known about the timing of these changes. Understanding temporal patterns in recovering microbial communities is an important part of improving how we assess and manage reconstructed ecosystems. We assessed patterns of community-level microbial diversity and abundance in corn and prairie plots 2 to 4 years after establishment in agricultural fields, using phospholipid fatty acid biomarkers. Principal components analysis of the lipid biomarkers revealed differing composition between corn and prairie soil microbial communities. Despite no changes to the biomass of Gram-positive bacteria and actinomycetes, total biomass, arbuscular mycorrhizal fungi biomass, and Gram-negative bacteria biomass were significantly higher in restored prairie plots, approaching levels found in long-established prairies. These results indicate that plant-associated soil microbes in agricultural soils can shift in less than 2 years after establishment of perennial grasslands.

## Introduction

Soil microbial communities are a vital component of terrestrial ecosystems because of their key roles in nutrient cycling [1], modification of plant community composition [2], regulation of plant productivity [3], and decomposition of organic matter [4]. This is particularly true in natural or minimally-managed ecosystems, where nutrient limitations and other considerations cause plants to develop dense below-ground associations with soil microbes [5], [6]. The recovery of soil microbial communities is a necessary element in returning a restored ecosystem to its desired structure and function [7], [8], but this recovery often lags behind changes in land use and land management [9], [10]. Thus, it is beneficial to know how quickly and thoroughly soil microbial communities can adjust in response to restoration-minded changes to the plant community and its management.

To a large extent, plant-microbe interactions in natural systems are driven by nutrient limitation, which leads plants to rely on relationships with bacteria and fungi for most of their mineral nutrition [5], [6], [11]. Symbiotic relationships are particularly important in the rhizosphere, where plants exude labile carbon compounds to stimulate the growth of microbes that can supply limiting resources such as nutrients and water [1], [12]. As a result, natural systems often have denser microbial communities than heavily managed systems [6], [13], [14]. Intensive agricultural management disrupts these relationships through exogenous nutrient application and other management interventions. Fertilization can allow plants to focus resources on fruit and grain production, often to the detriment of their microbial associations [11]. Physical disruption of the soil through tillage can increase plant productivity by liberating nutrients previously tied up in soil organic matter [12], but also damaging hyphae-producing microorganisms such as fungi and actinomycetes [6]. These changes in plant resource allocation come at the expense of soil organic matter and microbial biomass [6], [13]. Microbial functional groups that associate with plants and their rhizosphere are often most heavily impacted by these changes to plant-microbe relationships [15]

Classifying microbes into physiologically-based functional groups can obscure key taxonomic and functional distinctions, but in soil ecology there are some useful general properties that can be associated with certain groupings. For instance, arbuscular mycorrhizal (AM) fungi are particularly important in grassland communities [1], [6], where they colonize the roots of host plants and form hyphal networks that explore and exploit the surrounding soil to make limiting nutrients more available to their host. In some systems, a symbiotic relationship with secondary plant species can make them more competitive, while in other systems associations with dominant taxa can further exclude less competitive species [1]. Similarly, legume-associated nitrogen fixing bacteria are part of the Gram-negative (Gm-) functional group, and can significantly alter nutrient dynamics. In contrast, Gram-positive (Gm+) bacteria are often free-living and less responsive to land use and land cover changes [16]. Despite the limitations of these functional classifications, this approach can be a useful means of describing coarse changes to microbial communities during land use and land cover changes.

Phospholipid fatty acids (PLFAs) are frequently used to estimate functional group biomass in soils [17]. Because of their relatively high abundance and wide chemical variety in soil, PLFAs can be used as biomarkers of the dominant taxa comprising microbial communities [18]. Specific signature fatty acids can be used to determine the biomass of groups such as Gm+ and Gm- bacteria, AM and saprotrophic fungi, and actinomycetes [8], [12], [15], [18]. PLFAs can also be used to assay the physiological status of the microbial community [12] and have been used to gauge responses to a number of soil characteristics such as heavy metals, pH, and water availability, as well as genetically modified crops [19]. PLFAs decompose rapidly after cell death, allowing them to approximate the active portion of the microbial community at the time of sampling [12], [18]. This can be extremely helpful for studying the temporal complexity of plant-microbe interactions, given the high rates of dormancy observed among soil microorganisms [20]. PLFAs can more accurately characterize soil microbial community structure and detect community shifts than other current methods such as whole-cell fatty acid extractions [18]. The PLFA method is thus well suited to characterizing how soil microbial functional groups change in response to land use and land cover changes.

We used a bioenergy cropping systems experiment to study how the land use and land cover changes of prairie restoration affect the soil microbial community. We tracked total microbial biomass, functional group biomass, and overall lipid composition in matched continuous corn and prairie plots 2–4 years after the prairie's establishment on formerly cropped land. Further, we were interested in whether and how these systems diverged over time during the establishment phase of these perennial crops.

## Methods

### Study site and experimental design

Field sampling was conducted on land rented to the DOE-Great Lakes Bioenergy Research Center (GLBRC)—which is located on the Arlington Agricultural Research Station (ARL, Arlington, WI, 43°18′10 N, 89°20′40 W)—during mid-August in 2010 through 2012. As this sampling was done by GLBRC personnel and was part of core GLBRC data generation efforts, no specific permits were required. This research did not involve endangered or protected species. The site is predominantly Plano silt loam, which is known for high levels of organic matter, high cation exchange capacity and high agricultural productivity [16]. Historically, about 2/3 of the study area (blocks 1 through 3) had been involved in annual crops research, while the remainder (blocks 4 and 5) produced forage for use at ARL. The entire site was managed for a typical dairy rotation of forage crops and received inputs of urea or anhydrous ammonium. From 2005 to 2008, blocks 1 through 3 were under a hayfield mix of alfalfa and orchardgrass (*Medicago sativa* L. and *Dactylis glomerata* L.), while blocks 4 and 5 were planted to corn (*Zea mays* L). Both areas were managed using UW Extension-recommended nutrient management and “no till” conservation practices, with blocks 4 and 5 fertilized using swine effluent. Primary tillage took place in the fall of 2007 for blocks 4 and 5 and in spring 2008 for blocks 1 through 3. The entire study site received secondary tillage in spring 2008 after which corn and prairie plots were established. The corn plots have been fertilized with 28% UAN fertilizer following UW Extension recommendations; insecticides and herbicides were used for control as needed. Plots were under no-till management and were harvested with a 4.6-m combine with 60 to 70% stover removal. The prairie plots were seeded with a mixture of 18 native prairie species (listed in Table 1) in June 2008. Canada wildrye (*Elymus canadensis*) yellow coneflower (*Ratibida pinnata* Barnhart.) and big bluestem (*Andropogon gerardii* Vitman.) were the most abundant species 2010 through 2012 (LG Oates, personal communication). The prairie plots were planted with a Brillion planter and were not fertilized. During the first year they were mowed twice for weed suppression and each year were harvested in autumn to a residual stubble height of 15 cm within two weeks after the first killing frost.

**Table 1:** Plant functional group and the 18 species sown in the prairie plots.

Functional group	Species
Graminoids	*Andropogon gerardii*
	*Elymus canadensis*
	*Koeleria cristata*
	*Panicum virgatum*
	*Schizachyrium scoparium*
	*Sorgastrum nutans*
	
Legumes	*Baptesia leucantha*
	*Desmodium canadense*
	*Lespedeza capitata*
	
Early Forbs	*Aneomone canadensis*
	*Asclepias tuberose*
	*Rudbeckia hirta*
	
Mid Forbs	*Monarda fistulosa*
	*Ratibita pinnata*
	*Silphium perfoliatum*
	
Late Forbs	*Aster novae-angliae*
	*Solidago rigida*
	*Solidago speciosa*

### Soil sampling

In mid-August of each year in the study period we took five cores (15 cm depth, 37-mm diameter) of bulk soil from each plot. Cores were collected in a staggered transect covering about ¼ of the plot. They were transported on ice to the laboratory and within 24 h sieved to 2 mm. Sieved samples were frozen within 48 h of collection and subsequently freeze-dried for storage at -20°C.

### Microbial community analysis

Soils collected in 2008 from this experiment were assayed with PLFA to estimate microbial biomass and composition by Liang *et al*. [16], which provided us with baseline data to compare to 2010 through 2012. In addition, PLFA data from soils taken in 2008 from 9 sites across southern Wisconsin where prairies that had been restored for more than 5 years were used to benchmark microbial biomass abundances expected under undisturbed settings [6].

In 2013, we used a combined procedure of phospholipid fatty acid (PLFA) and fatty acid methyl ester (FAME) extraction to assay the microbial community. Using a modified lipid extraction technique initially described by Balser and Firestone [21] lipids were quantified and identified. We extracted 3 g of homogenized freeze dried soil with a 2.7-ml phosphate buffer in 3.0 ml chloroform and 6.0 ml methanol. To analyze the extracts we saponified the fatty acids by adding sodium hydroxide, followed by strong acid methanolysis. Extracts were analyzed on a Hewlett-Packard Agilent 6890A gas chromatograph (Agilent Tech. Co., Santa Clara, CA) equipped with a 25-m×0.2-mm×0.33-µm Agilent Ultra-2 (5% phenyl)-methylpolysiloxane capillary column (Hewlett Packard, Palo Alto, CA) and flame ionization detector. MIDI's EUKARY method database was used to identify fatty acids. We did not use internal standards as described by Balser and Firestone [24]. Indicator lipids were used for detecting functional group biomass (Table 2).

**Table 2:** Indicator lipids for microbial functional groups.

Functional group	Indicator lipid
Actinomycetes	10Me 16:0
	10Me18:0
Gram positive bacteria	a15:0 *
	i15:0 *
	a17:0 *
	i17:0 *
Gram negative bacteria	16:1ω7c ^†^
	17:0cy *
	18:1ω5c
	18:1ω7c
	19:0cy
AM fungi	16:1ω5c
General fungi	18:1ω9c
	18:2ω6,9c

Lipids marked with * were used to calculate stress ratios; ^†^denotes a precursor.

### Statistical analysis

Statistical analysis was carried out in R (version 3.0.3) [22] using the packages ‘nlme’ (version 3.1-109) [23], ‘lsmeans’ (version 1.06-05) [24], and ‘vegan’ (version 2.0-7) [25]. We used the function ‘lme’ to construct linear mixed effects models using block as a random effect to account for variation in land use history. For each functional group and total biomass we separately tested different variance structures allowing for unequal variances among crops, years, or both (i.e., ‘weights  =  varIdent’) with log-likelihood ratio tests used to select between nested models. Ratios were log-transformed for analysis and back transformed to give geometric means. Significance of pairwise differences among samples was calculated using the ‘lsmeans’ function with the Tukey-Kramer multiple comparison correction. Principal components analysis was conducted using the ‘rda’ function with arcsine-square root transformed mole percent data.

## Results and Discussion

### Microbial biomass

**Figure 1:**
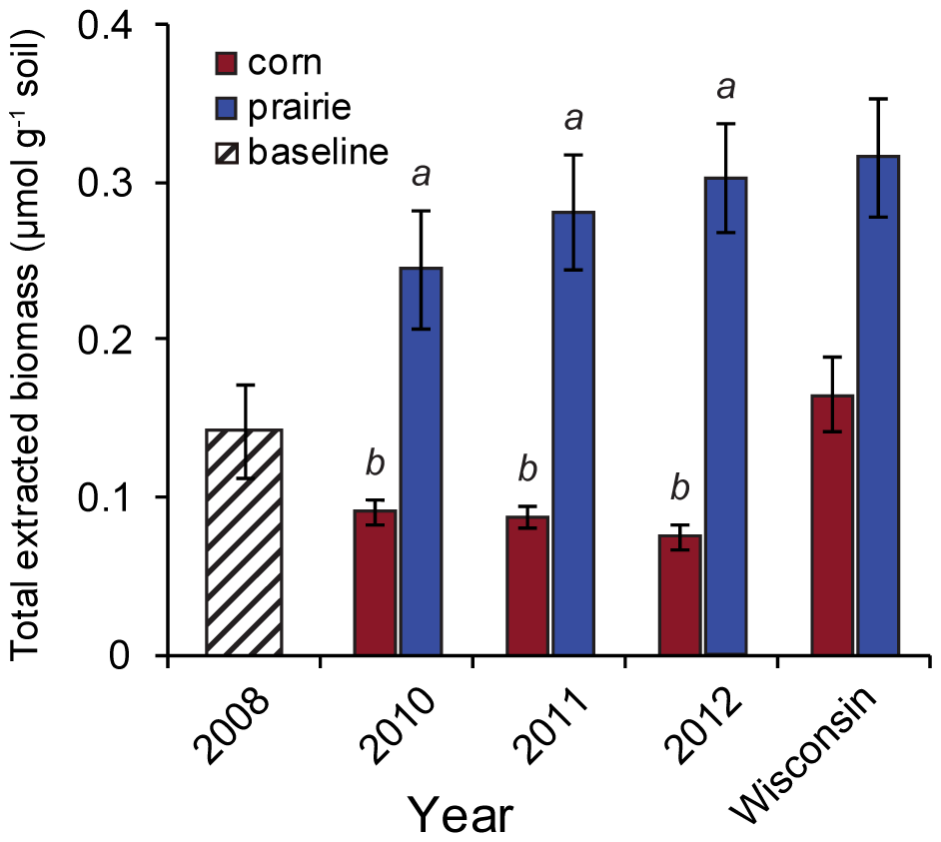
Total sample biomass (µmol/g) in corn and prairie sites. Error bars are +1 standard error. Baseline data adapted from Liang *et al*. [18], statewide Wisconsin data adapted from Liang *et al*. [12] based on observations from 2008. Bars are ±1 standard error. Groups sharing a letter are not significantly different at a *P*<0.05 significance level.

Natural systems are generally thought to have higher microbial biomass than intensively managed agricultural systems [12], [14]. At the time of establishment, the microbial biomass at our study site was in line with values obtained for corn fields in the general area (Fig. 1). By the second year after plot establishment, we were able to detect a significant difference in total microbial biomass between prairie and corn plots (0.24 vs 0.09 µmol g^−1^ soil; P<0.05). Microbial biomass remained higher in prairie plots than corn plots throughout the measurement period, although the difference did not change significantly over time (Fig. 1).

The difference in microbial biomass between natural and intensively managed agricultural systems is frequently attributed to mechanical disruption of fungal hyphae caused by tillage [6], or to the greater investment in roots and root exudates by native perennials relative to corn [6], [12], [14], [26]. As the corn system we studied was no-till, the rapid changes we observed were most likely driven by differences plant-microbe interactions between the systems. Indeed, the most dynamic changes occurred in functional groups where plant-associated microbes are heavily represented.

### Plant-associated microbes

**Figure 2:**
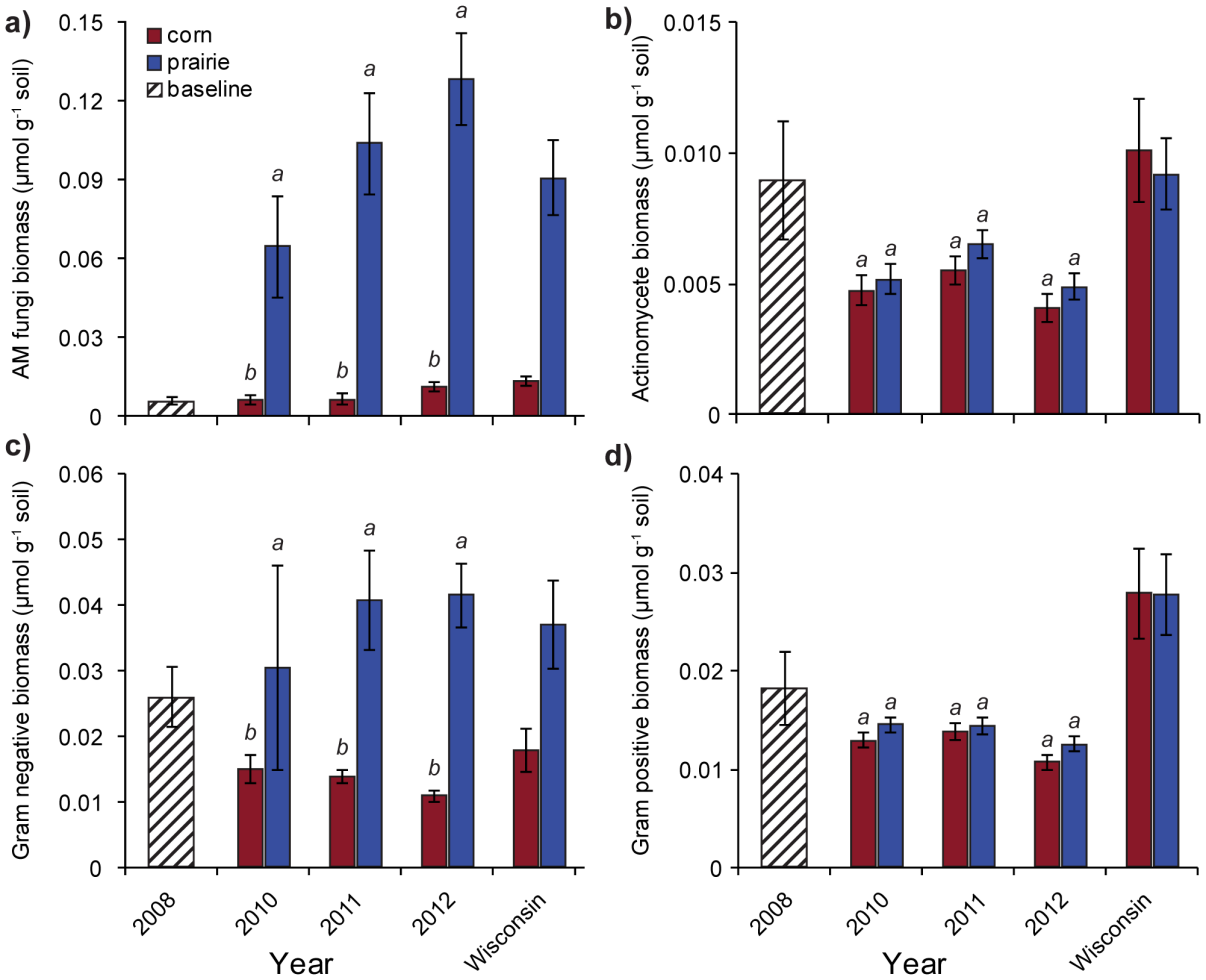
Absolute abundance of microbial functional group lipid biomarkers: (A) arbuscular mycorrhizal fungi; (B) actinomycetes; (C) Gram-negative bacteria; (D) Gram-positive bacteria. Baseline data adapted from Liang *et al*. [18], statewide Wisconsin data adapted from Liang *et al*. [12] based on observations from 2008. Bars are ±1 standard error. Groups sharing a letter are not significantly different at a *P*<0.05 significance level.

The strong associations formed between AM fungi and certain prairie plants can substantially impact ecosystem dynamics and plant community composition in natural systems [1]. Arbuscular mycorrhizal fungi were the microbial group that showed the greatest difference between corn and prairie fields in southern Wisconsin [6]. We observed a 20-fold increase in AM fungal biomass from 2008 to 2012 in prairie plots, while in corn, AM fungal biomass remained similar in 2012 (0.016 µmol g^−1^ soil) to the initial value (0.006 µmol g^−1^ soil) reported by Liang et al. [16] for 2008 (Fig. 2A). Across years, total fungal biomass was significantly higher in prairie plots compared to corn plots (0.043 vs 0.009 µmol g^−1^ soil; P<0.01). In the absence of tillage as an explanatory factor, this drastic change speaks to the capacity of native prairie plants to promote the growth of associated fungi in the absence of substantial exogenous nutrient inputs.

Like AM fungi, many taxa classified within the functional group of Gm- bacteria benefit from the nutrient rich environment provided by a plant rhizosphere. Total biomass for Gm- bacteria was also higher in the prairie than in the corn (0.005 vs 0.002 µmol g^−1^ soil; P<0.05; Fig. 2C). As we observed with AM fungal biomass, this change was visible from the second year, but did not change significantly over the study period. Interestingly, this was the sole functional group where the baseline biomass observed in 2008 was higher than the values observed for corn fields in the area (Fig. 2). Given that 3 of our 5 blocks had been in an alfalfa-soybean rotation prior to the establishment of this experiment [16], there could have been a relatively large residual population of legume associated Gm- bacteria in the soil at the start. This could have been recruited by the legumes in the prairie (Table 1), potentially accelerating the increase in Gm- biomass we observed in that system.

### Unassociated microbes

Undisturbed soils tend to have higher amounts of microbes associated with plants in the rhizosphere [5], [14], while unassociated microbes may be less affected by soil disturbance and found in both the rhizosphere and the bulk soil [6], [12], [26]. We did not see a difference in the amount of Gm+ bacteria in corn and prairie (0.01 vs 0.01 µmol g^−1^ soil; P>0.05; Fig. 2D) or actinomycete biomass (0.0048 vs. 0.0049 µmol g^−1^ soil; P>0.05; Fig. 2B); which are both typically associated with microbes usually found in the bulk soil that can thrive in more physically stressed environments [6]. Millard and Singh [14] indicated that these types of microbes should be more affected by land use and land cover on decadal time scales. Our plots were under the same land use history use for many years, which may be why we observed little difference unassociated microbial taxa between cropping system treatments. Because Gm+ bacteria are less affected by land use and land cover shifts, their total abundance remained stable in the prairie plots while their relative abundance decreased with the increase in other functional groups (5.1 mol% vs 14.8 mol%).

Young prairies tend to have higher relative abundance of AM fungi, but they may see an increase in relative abundance of actinomycetes as they mature [12]. Our prairie plots had relative abundance values of 41.5 mol% for AM fungi and 1.7 mol% for actinomycetes. As the microbial community continues to look more like an established prairie, we would expect actinomycetes to become more prevalent [12]. On the other hand, Huang *et al.* [27] did not see a large increase in the relative abundance of actinomycete biomass in a 78-year grassland chronosequence. For both corn and prairie, actinomycete biomass was significantly higher in 2011 (corn: 0.0056 µmol g^−1^ soil, prairie: 0.0066 µmol g^−1^ soil) than 2012 (corn: 0.0041 µmol g^−1^ soil, prairie: 0.0049 µmol g^−1^ soil, Fig. 2B). This interannual variability may be weather-related reflecting the moderate to severe drought experienced in southern Wisconsin during the 2012 growing season.

### Soil storage considerations

Our results should be interpreted with the caveat that these soils were stored frozen and freeze-dried for over a year prior to PLFA extraction. Studies have demonstrated that frozen storage can reduce the mass of PLFAs recovered from soils [28], although the sensitivity seems lower for arable or formerly agricultural land [29]. Very few studies seem to have looked at the effect of storage time, although Wallenius *et al*. [30] found no change in PCR amplicon length heterogeneity profiles between soils stored frozen for 1 and 16 weeks. While storage-driven PLFA mass loss may explain the reduced total biomass in corn plots, and actinomycete and Gm+ biomass in both systems relative to the 2008 baseline, we observed no changes in these values across years, as would be expected if there were progressive degradation. Given that we found within-year differences between systems and that these differences remained consistent across years, it seems highly unlikely that our results and their interpretation are tainted by long-term frozen storage, although caution should naturally be exercised when comparing the absolute biomass values we present here to values obtained with different soil storage, handling, and extraction methods.

### Fungal and bacterial dynamics

Fungal to bacterial biomass (F:B) ratios are often used to understand the functional importance of microbial community change [12], [26], [31] and an increase in F:B can be indicative of a shift towards an undisturbed soil community [7], [14]. In our soils, F:B was higher in the prairie (geometric mean 1.01) than the corn plots (geometric mean 0.71), but did not differ significantly from year to year. This was primarily driven by the large increase in AM fungal biomass, rather than a decrease in bacterial biomass. Allison *et al*. [12] observed similar values in recently established prairie plots and also noted the driving effect of AM fungi. Similar results were found by Hedlund [26] who observed a significant microbial community change led by AM fungal biomass increase within two years of an agricultural field being sown with prairie species, but little directional change in subsequent years.

### Stress indicators

PLFAs can be used to indicate the physiological state of the microbial community as well as its composition [32]. Microbes are affected by environmental factors and can change the composition of their cell membranes in response to the environment [32]. Using ratios of certain PLFAs and their precursors we can assess the effect of nutrient and environmental stress on microbes producing these lipids. This analysis has been used to indicate a variety of stressors such as starvation, drought, and heavy metal pollution [32].

The increase in cyclopropyl fatty acids (cy17:0) relative to their precursor (16:1ω7c) is a commonly used method for detecting Gm- bacteria starvation stress where a cyclopropyl:precursor ratio >0.1 is indicative of environmental stress [32]. Higher ratios are often found in agricultural fields, implying the microbial community is under environmental stress [12], [32]. In our study, the only system that had a (cy17:0)/(16:1ω7c) ratio <0.1 was prairie in 2012 (0.09) indicating that corn (0.25, 0.31, 0.29, in 2010 through 2012, respectively) was stressed in all three years, while prairie was similarly stressed physiologically for 2010 (0.15) and 2011 (0.12). The lower ratio in prairie for 2012 may be indicative of more preferable conditions as time passed between establishment of prairie plots from annual cropping systems. While this may also be attributable to storage effects, the corn system did not change in the same fashion.

**Figure 3:**
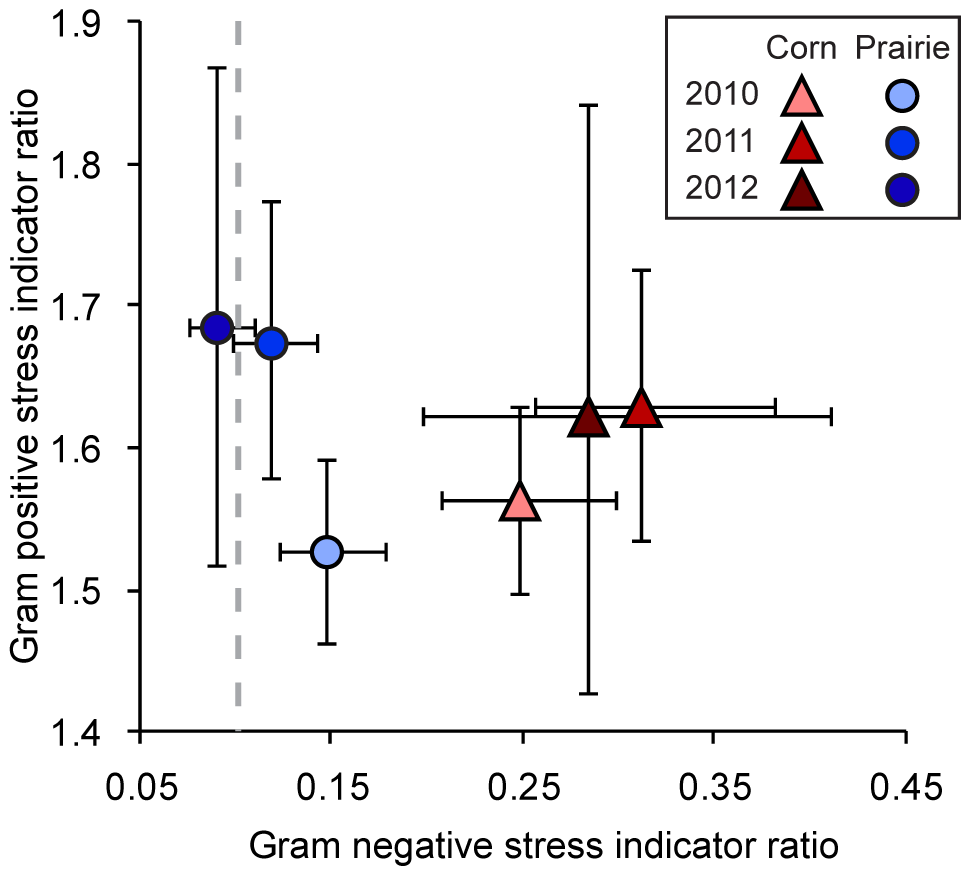
Bacterial metabolic stress indicators. Horizontal axis indicates 17:0cy:16:1ω7c, vertical axis indicates (i15:0+i17:0):(a15:0+a17:0) based on molar masses. In both cases greater values indicate increasing stress. Dashed line indicates the upper bound for an unstressed Gram negative community. Values are geometric means ±1 standard error.

Another ratio that typically indicates nutrient and environmental stress is the ratio of lipids (17:0iso+15:0iso)/(17:0anteiso+15:0anteiso). This differs from the above ratio in that it is indicative of Gm+ bacteria. We did not find significantly different ratios for the Gm+ stress indicator between corn (1.62) and prairie (1.68) in 2012 or any other year (Fig. 3).

### Principal components analysis

**Figure 4:**
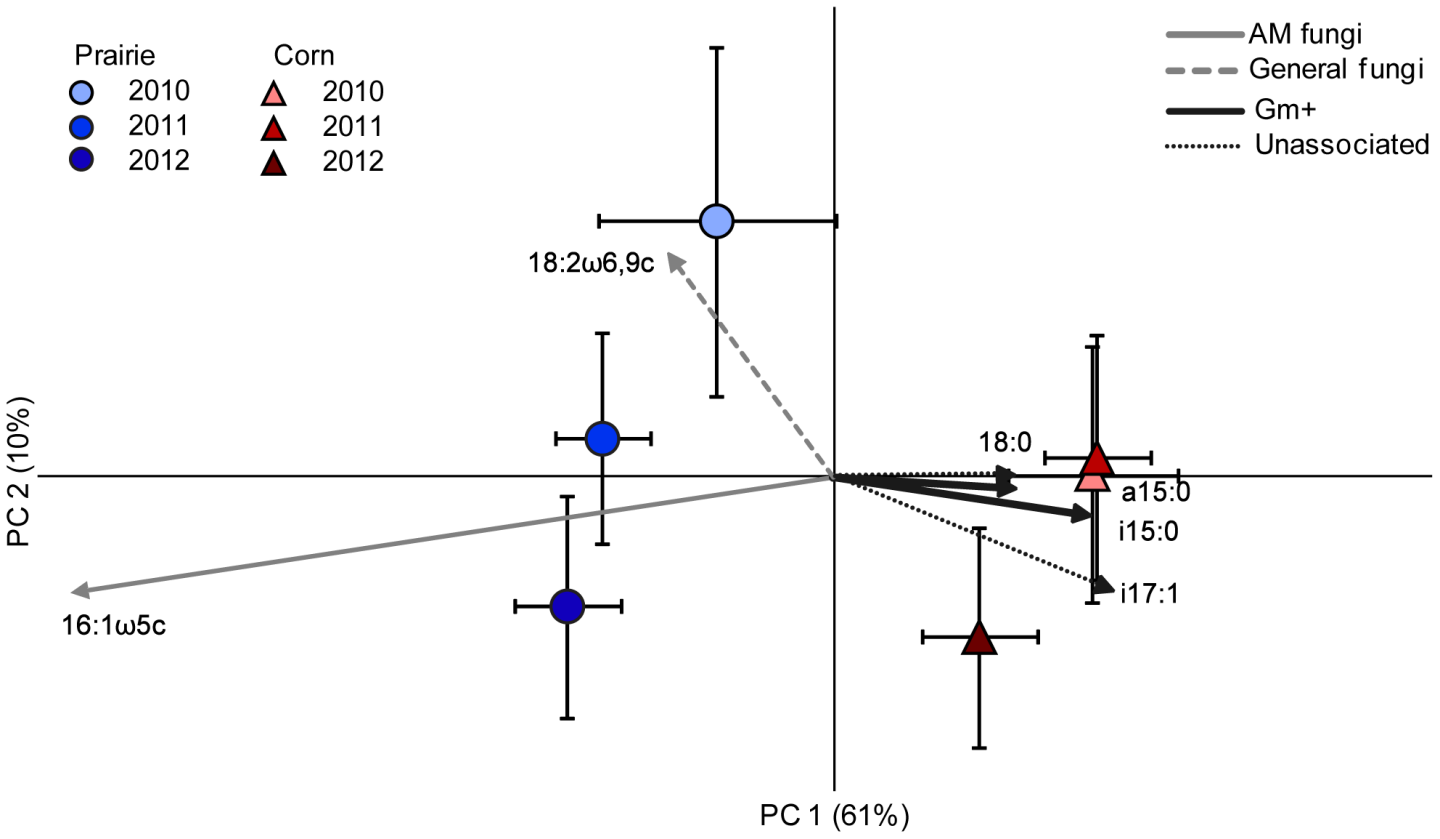
Principal component analysis of soil microbial community composition based on phospholipid fatty acid profiles. Plotted values are group means ±1 standard error. Vectors indicate loadings of the six most relevant individual lipids, separated by associated microbial functional groups.

While our analysis emphasized microbial functional groups, PLFA data can also be used to evaluate the overall similarity of microbial communities [15], [32], [33] using principal components analysis (PCA). The first PCA axis was very strong accounting for 61.3% of the variation in our PLFA matrix and separating prairie and corn treatments (Fig. 4). This axis largely recapitulated our earlier results, showing the relative abundance of Gm+ bacteria and AM fungi, as well as some unassociated lipids, are what separated corn from prairie. Our second axis was much weaker, accounting for only 10.7% of the variation and showing slight separation among years based on abundance of fungi, unassociated microbes, and, to a lesser extent, AM fungi. From this analysis, we see a clear distinction between corn and prairie and an inkling that the prairie is changing over time as well, although once again some of this may be due to storage effects (Fig. 4).

### Methodological considerations

Understanding what microbial communities influence and how they are influenced by land use and land cover is difficult because of the interdependence of ecosystem components and variability of environmental factors, as well as the tools we use to understand them. The variety of methods available and the lack of precision among the techniques may cause different methods to converge on distinct conclusions [1], [34]. For instance, Suleiman *et al*. [3] used 16S ribosomal subunit sequencing to test the effects of removing plant cover via deforestation of a small area inside a forest. They showed 69% of the operational taxonomic units (OTUs) were not detectably different between the forest and the area where vegetation had been removed eight years after transition from forest to grassland, and concluded that microbial function did not suffer in response to land use change. If we had used a similar approach our results may have been drastically different.

### Conclusions

Our results illustrate that the microbial community can shift rapidly (within 2 years here) when perennial prairie is established on formerly cropped lands. We observed a rapid recovery of certain plant-associated microbes and an increase in total microbial biomass. Our findings suggest the latent microbial community in historically agricultural former prairie soils may be sufficient to support rapid reestablishment of key microbial functional groups.
